# Improved Motor Nerve Regeneration by SIRT1/Hif1a-Mediated Autophagy

**DOI:** 10.3390/cells8111354

**Published:** 2019-10-30

**Authors:** David Romeo-Guitart, Tatiana Leiva-Rodriguez, Joaquim Forés, Caty Casas

**Affiliations:** 1 Institut de Neurociències (INc) and Department of Cell Biology, Physiology and Immunology, Universitat Autònoma de Barcelona (UAB) & Centro de Investigación Biomédica en Red sobre Enfermedades Neurodegenerativas (CIBERNED), Bellaterra, E-08193 Barcelona, Spain; romeoguitart@gmail.com (D.R.-G.); tatileivarodriguez@gmail.com (T.L.-R.); 2 Hand and Peripheral Nerve Unit, Hospital Clínic i Provincial, Universitat de Barcelona, Barcelona, Spain Biotech, S.L, E-08193 Barcelona, Spain; JFORES@clinic.cat

**Keywords:** peripheral nerve injury, axonal regeneration, motoneuron, autophagy, SIRT1/Hif1α, NeuroHeal

## Abstract

Complete restoring of functional connectivity between neurons or target tissue after traumatic lesions is still an unmet medical need. Using models of nerve axotomy and compression, we investigated the effect of autophagy induction by genetic and pharmacological manipulation on motor nerve regeneration. ATG5 or NAD^+^-dependent deacetylase sirtuin-1 (SIRT1) overexpression on spinal motoneurons stimulates mTOR-independent autophagy and facilitates a growth-competent state improving motor axonal regeneration with better electromyographic records after nerve transection and suture. In agreement with this, using organotypic spinal cord cultures and the human cell line SH-SY5Y, we observed that the activation of SIRT1 and autophagy by NeuroHeal increased neurite outgrowth and length extension and that this was mediated by downstream HIF1a. To conclude, SIRT1/Hifα-dependent autophagy confers a more pro-regenerative phenotype to motoneurons after peripheral nerve injury. Altogether, we provide evidence showing that autophagy induction by SIRT1/Hifα activation or NeuroHeal treatment is a novel therapeutic option for improving motor nerve regeneration and functional recovery after injury.

## 1. Introduction

Peripheral nerve injury (PNI) is caused by traffic accidents, lesions at home, or at the workplace, which may result in a partial or total loss of motor function or sensory perception [1]. PNI directly affects about approximately 13–23 people per 100,000 per year, aged 20–40 years, resulting in an important economic cost in the healthcare of developed countries, mostly due to sick leave and the consequent loss of production [2,3,4]. Surgical repair, decompression, lysis, and functional exercise are important in recovering the function of a peripheral nerve promoting regeneration and maintaining muscle mass [5]. Nevertheless, functional recovery is often not satisfactory despite the intrinsic capability for regeneration of injured axons or reinnervation by collateral branching of undamaged axons in the vicinity of the target [1]. Currently, at clinics, there is no coadjuvant treatment in use to promote nerve regeneration. For this purpose, our group has recently proposed a promising compound named NeuroHeal [6,7]. It was designed using a network-centric approach and artificial intelligence and proved to be neuroprotective and accelerate nerve regeneration in several rodent models of axotomy [6,7]. NeuroHeal is formed by a combination of two FDA-approved drugs: acamprosate and ribavirin. One of the key nodes of the synergic mechanism of action of NeuroHeal in neuroprotection is the NAD^+^-dependent deacetylase sirtuin-1 (SIRT1), a widely described sensor of cellular stress [8,9,10]. However, the underlying mechanisms involved in nerve regeneration are not known yet. 

We have recently demonstrated that NeuroHeal induces fine-tuned pro-survival macroautophagy (hereafter, termed autophagy) to neuroprotect axotomized neonatal motoneurons from apoptosis [manuscript in revision]. [11]. Autophagy is a catabolic process essential for sustaining the living and homeostasis of the cells, characterized by the presence of de-novo formed autophagic vesicles (autophagosomes) with superfluous or potentially dangerous cytoplasmic material that is delivered to lysosomes for degradation [11]. It can be triggered by several different stimuli, including the activation of SIRT1 [12]. Because of their polarized morphology, neurons face special challenges when recycling cellular components through autophagy in dendrites and distal regions of axons. The canonical formation of the autophagosome involves different steps including induction, autophagosome formation, the fusion of the autophagosome with the lysosome, and cargo degradation, followed by the release of breakdown products into the cytosol. Autophagy involves a group of highly conserved genes, first found in yeast, termed autophagy-related genes (ATGs). In cells undergoing autophagy, phagophore formation initiates after the Unc-51 like autophagy activating kinase 1 (ULK1) activation, and its elongation is regulated by two ubiquitin-like reactions: the first leading to the formation of the complex ATG12-ATG5-ATG16L1; and the second involving the conjugation of the microtubule-associated protein light chain 3 (MAP-LC3/ATG8/LC3) to phosphatidylethanolamine (PE) at the autophagosome membrane to form autophagosome-associated LC3-II. Once the autophagosome is formed, it acquires the ability to bind autophagic substrates and/or proteins that mediate cargo selectivity (including p62). Autophagosomes mobilize toward lysosomes along microtubules; then, the outer membrane of the autophagosome fuses with the lysosome to form an autolysosome. Proper function and integrity of the lysosome are essential for successful fusion to occur. Degradation of the inner membrane and autophagosome content, including LC3-II, occurs in autolysosomes and relies on lysosomal hydrolases. Mice lacking Atg5, Atg7, and FIP200, specifically in the central nervous system, show neurological and behavioral defects, axonal degeneration, and neuronal loss [13]. These findings highlight the importance of a continuous clearance of cytosolic proteins through basal autophagy, to prevent the accumulation of abnormal proteins, which may impair neuronal function.

From the middle mid-1960s, nerve crush studies on the rat sciatic nerve have established the presence of autophagic vesicles within the nervous system [14]. The implication of autophagy in nerve regeneration is still a matter of investigation. Huang and collaborators (2016) suggested that m-TOR dependent autophagy induction in Schwann cell biology at the nerve stimulates nerve regeneration [15]. Stimulation of autophagy after spinal cord injury lesion attenuated axonal retraction and promotes regeneration of descending axons in the long-term by stabilizing microtubules [16]. However, there is a lack of studies investigating the gap in understanding whether autophagy induction may be relevant within the motoneurons (MNs), when they shift from an active, electrically transmitting state back to a silent, growth-competent state that occurs after nerve injury [17]. Thus, it seems likely that induction of autophagy may be beneficial for motor nerve regeneration, in particular through activation of SIRT1; herein, we explore these issues.

## 2. Materials and Methods

### 2.1. Surgical Procedures

All the experiments involving animals were approved by the Ethics Committee of our institution and followed the European Community Council Directive 2010/63/EU. Sprague-Dawley rats were kept under standard conditions of light, temperature, and feeding and at 12 weeks of age, we performed the surgical procedures. We deeply anesthetized rats with a cocktail of ketamine/xylazine 0.1 mL/100 g administered intraperitoneally (i.p.) and prepared the animal for surgery. For crush injury, the sciatic nerve of the right hindlimb was exposed ad mid-high and compressed with fine forceps (3 times of 30”) at 90 mm from the third toe [7]. For the cut and suture, we dissected the sciatic nerve as previously described, transected it at 90 mm from the third toe and immediately repaired it with a fascicular suture (10-0, Ethicon). Following all surgeries, the wound was closed, disinfected, and the animals were allowed to recover in a warm environment. For the adeno-associated viral (AAV) injected rats, surgery was performed three weeks after injection to ensure an optimal gene expression. 

The ventral root avulsion (RA) and delayed root reimplantation (RE) were carried out as previously described [6]. Briefly, we performed laminectomy at T11 vertebra to release the L3–L6 ventral roots from the meninges, and we detached them separately from the spinal cord with the help of a hook. In addition, we introduced the four injured roots into a silicone tube, repaired the wounds, and allowed the animals to recover. Two weeks after RA, we anesthetized the animals and checked by electrophysiological tests for complete muscle denervation. We localized the silicone tube, dissected the injured ventral roots, and inserted underneath them the corresponding spinal cord segment. To ensure maintenance of the reimplanted roots within the spinal cord, we opposed the paraventral muscles to the spinal cord and close the wounds.

For the hypoglossal model, wild type female C57BL/6 (Charles River Laboratories, Wilmington, MA, USA) mice, aged 2 months and weighing an average of 24.92 ± 1.66 g (Animal Service, Universitat Autònoma de Barcelona (UAB)), were maintained under standard conditions of temperature and light and fed with food and water ad libitum. Surgical procedures were performed under anesthesia with ketamine (90 mg/kg, intramuscularly [i.m.]) and xylazine (10 mg/kg, i.m.). We carried out axotomy of the hypoglossal nerve as described elsewhere [18]. Briefly, the right digastric muscle was opened using blunt-end dissection with a pair of scissors, and the right hypoglossal nerve was exposed. We transected the nerve with a pair of scissors at the proximal side of the hypoglossal nerve bifurcation and removed 3 mm from the distal stump. Finally, we separated the nerve stumps to avoid spontaneous axon regrowth. The muscle was sutured, and the wound closed by planes and disinfected with povidone-iodine. The animals were allowed to recover in a warm environment.

### 2.2. Construction, Purification, and Infection with Recombinant Adeno-Associated Vectors

SIRT1 cDNA and ATG5 cDNA were cloned into NheI and XhoI sites between the inverted terminal repeats (ITRs) of AAV2, under the regulation of the cytomegalovirus (CMV) promoter and the woodchuck hepatitis virus responsive element (WPRE) [19]. The AAV2/rh10 vector was generated as previously described [20] by triple transfection of HEK 293-AAV cells (Stratagene) with branched polyethylenimine (PEI; Sigma) with the plasmid containing the ITRs of AAV2, the AAV helper plasmid containing Rep2 and Cap for rh10 (kindly provided by JM Wilson, University of Pennsylvania, Philadelphia, USA) and the pXX6 plasmid containing the helper adenoviral genes [21]. Recombinant vectors were clarified after benzonase treatment (50 U/mL, Novagen) and polyethylene glycol (PEG 8000, Sigma) precipitation. Vectors were purified using an iodixanol gradient at the Vector Production Unit of UAB (http://sct.uab.cat/upv), following standard operating procedures. Viral genomes per ml (vg/ml) were quantified with PicoGreen (Invitrogen, Carlsbad, CA, USA).

We performed intrathecal injection of 4 × 10^10^ viral genomes under ketamine/xylazine-anesthetized animals using a Hamilton syringe with a 33-gauge needle. For intrathecal injection, the vertebral column was exposed after muscle dissection at L3–L4 vertebrae, and 10 μL of viral vectors were slowly injected into the cerebrospinal fluid between vertebras. We introduced the needle and correct intrathecal placements were confirmed by the animal tail flick. The needle was fixed in the injection site for 10 s to avoid fluid retraction and the wound was sutured [7].

### 2.3. Drug Administration

NeuroHeal (NH) is composed of acamprosate calcium (Aca) and Ribavirin (Rib) compounds [7]. Aca, Rib, Ex-527, and nicotinamide (NAM) (Sigma-Aldrich, Saint Louis, MO, USA), and 3-Methyladenine (3MA) (Tocris) for in vitro studies were diluted in sterile H_2_O or DMSO. Aca, Rib, Ex-527, and NAM were added at a final concentration of 1 μM, 55 μM, 10 µm, 5 mM, and 10 μM, respectively, and were mixed with the culture medium.

In vivo treatment with NH consists of Aca (Merck) and Rib (Normon) pills grounded into a fine powder and dissolved in drinking water at 2.2 mM and 1 mM, respectively. In some experiments, NAM was dissolved and added also in the drinking water at 5 mM. We changed the tap water every 3 days and freshly added the drug treatment on a daily basis. DMOG (Tocris), dissolved in DMSO, was injected daily at 20 mg/kg.

### 2.4. Electrophysiological Test and Functional Assessment

For electrophysiological evaluation, rats were anesthetized with ketamine/xylazine (100:10 mg/kg weight, i.p.) at different times post-injury as described before [7]. Briefly, the sciatic nerve was stimulated by transcutaneous electrodes placed at the sciatic notch by single pulses (20 µs), and the compound muscle action potential (CMAP) was recorded by placing electrodes on the tibialis anterior (TA), gastrocnemius, and plantar interosseous muscles. Stimulus intensity was applied gradually until reaching the supramaximal stimulus, which corresponds to the maximum CMAP amplitude. The evoked action potentials were displayed on a storage oscilloscope (Synergy Medelec, Viasys HealthCare) at settings appropriate to measure the amplitude from baseline to peak and the latency to the onset after every stimulus. For the functional analysis of locomotion, we painted the plantar surface of rat hind paws with acrylic paint and allowed the rat to walk along a corridor onto a white paper. Footprints from operated and intact paws were analyzed by measuring the print length (PL), the distance between the 1st and 5th (TS) or 2nd and 4th (IT) toes with a precision device. The three parameters were combined to obtain the sciatic functional index (SFI) which quantifies the changes in walking pattern (0 for uninjured; −100 for maximally impaired gait) [22]. After testing, animals were allowed to recover in a warm environment. 

### 2.5. Spinal Organotypic Culture (SOCs) on Collagen 3D Matrix

We prepared spinal organotypic cultures as previously described [23]. In summary, we prepared collagen solution at 3 mg/mL by mixing: rat tail collagen type I (Corning, Wiesbaden, Germany), phosphate-buffered saline (PBS) (Sigma-Aldrich), sodium bicarbonate at 0.3 mg/mL, and 10X basal Eagle’s medium (Gibco, Grand Island, USA). We deposited 30 μL-single drops of collagen in 24-well Petri dishes pre-treated with poly-D-Lysine (Sigma-Aldrich) and kept them in the incubator for 1 h at 37 °C and 5% CO_2_ to induce collagen gel formation. Thereafter, we extracted lumbar spinal cord sections from 7 day old Sprague-Dawley rats, placed them in 30% glucose cold Gey’s balanced salt solution (Sigma-Aldrich) and removed the meninges and nerve roots. Spinal cords were cut into 350 μM–thick slices and were placed onto collagen droplets. After 30 min in the incubator, slices were covered with 30 μL of the same collagen solution mentioned above; following 30 min at 37 °C for collagen polymerization, we added Neurobasal (Life Technologies) culture medium supplemented with B27 (Life technologies), glutamine, and penicillin/streptomycin (Sigma-Aldrich). 

One day after culture, we removed the media and re-applied the same media combined with the different treatments: H_2_O or DMSO as a vehicle, NH, NH + Ex-527, NH+NAM NH+3MA, DMOG, DMOG+3MA, and 3-MA. We then changed the medium at 3 days post culture. After 4 days of treatment, we removed the media, post-fixed the spinal cord slices with cold 4% paraformaldehyde (PFA) solution for 1 h, washed them with Tris-buffered saline (TBS) several times, and incubated slices with primary antibodies for 48 h at 4 °C. For neurite growth analysis the primary antibody was an anti-mouse RT97 (1:200; Hybridoma Bank, USA). After washing with 0.1% Tween 20 in TBS, we incubated the spinal cord slices with a donkey-conjugated Alexa 594 anti-mouse antibody (1:200; Life Technologies) overnight at 4 °C, counterstained with DAPI, washed slices and mounted slides with DPX (Sigma-Aldrich).

We took sequential microphotographs with an Olympus BX51 (Olympus) fluorescence microscope attached to a DP73 camera (Olympus DP50) and merged images with Adobe Photoshop CS3 (Adobe System, USA) to obtain the entire spinal cord slice bodies with their neurites. To analyze neurite growth and length, whole culture images were analyzed with the help of the Neurite-J plug-in for ImageJ software [24]. The number of neurites for each intersection from the explant was calculated and compared between sets of cultures. 

### 2.6. Cell Culture

SH-SY5Y cells were grown in high-glucose Dulbecco’s Modified Eagle Medium (DMEM) supplemented with 15% fetal bovine serum (Sigma-Aldrich), and a 0.5× solution of penicillin/streptomycin (Sigma-Aldrich). Cells were kept in a humidified incubator at 37 °C under 5% CO_2_. For the treatments, we coated plastic plates (Thermo) with 10% collagen dissolved in Milli-Q water at 37 °C for 2 h. After removing this solution, we seeded the cells at a density of 2.5 × 10^5^ per cm^2^. For a differentiated phenotype, cells were grown in Neurobasal medium (Life Technologies) supplemented with B27 (Life technologies), 1 µM of retinoic acid (Sigma), and a 0.5× penicillin/streptomycin solution. After 3 days in culture without changing the medium, SH-SY5Y cells presented with a differentiated-like phenotype characterized by the presence of long neurite extensions. At this time, we added different drugs to the cells. The drugs, prepared at a ten-fold higher concentration than the concentration to be tested, were dissolved in DMEM to the desired concentration and used to replace the medium over cells. We used 1 mM DMOG (Tolcris) and 10 μM 3-MA (Merck-Millipore) unless otherwise stated. After 24 h, we fixed the cells with 4% PFA, rinsed twice with PBS, and stored at −20 °C or added blocking buffer containing PBS plus 0.3% (v/v) Triton X-100 and 10% fetal bovine serum. We incubated with the following primary antibodies: mouse anti-β-tubulin (1:500, Covance/BioLegend), mouse anti-HIF1α (1:500, Novus Biological), and rabbit anti-SIRT1 (1:200, Merck-Millipore) in 0.5× blocking buffer in PBS, at 4 °C overnight. The following day, after several washes with PBS plus 0.05% Tween-20, we incubated the cells on coverslips with Cy3- or Cy2-conjugated secondary antibodies (Jackson Immunoresearch); cells were counterstained with DAPI and coverslips were mounted with Mowiol. Images were taken under the same exposure times, sensitivities, and resolutions for each marker analyzed with the aid of a digital camera (Olympus DP50) attached to a fluorescence microscope (Olympus BX51).

For transfection experiments, we transfected 1 × 10^6^ cells with 1 µg, shRNAGFP (CSHCTR001-CH1, Tebu-bio), shRNA HIF1α (HSH008832-32-CH1, Tebu-bio), and SIRT1 using the Amaxa Nucleofector II TM (Lonza) and the Nucleofactor V kit (Lonza) following the manufacturer’s recommendations.

To analyze neurite growth, we took microphotographs at 20× magnification and culture images were analyzed with the help of ImageJ software. The growth of neurites was measured manually and compared between sets of cultures.

### 2.7. Tissue Processing for Histology

At end-stage, we euthanized the animals after dolethal injection (60 mg/kg i.p.) and transcardially perfused them with a saline solution of heparin (10 U/mL) followed by 4% paraformaldehyde in 0.1 M PBS buffer solution. In addition, we harvested L4–L5 spinal cord post-fixed them with 4% PFA for 1 h, and cryopreserved them in 30% sucrose solution. Serial spinal cord sections of 20 µm (20 series of 10 sections each) were obtained with the aid of a cryotome (Leica, Heidelberg, Germany) and kept at −20 °C until needed.

### 2.8. Immunohistochemistry and Image Analysis

Sections for each marker from different animals of each group to be analyzed together were immunolabeled simultaneously and the analysis was performed at the same time. We washed the spinal cord slices with TBS, blocked them with TBS-Glycine 0.1 mM during 1 h, and with blocking solution (TBS with 0.3% Triton-X-100 and 10% donkey serum) for 1 h at room temperature (RT). Next, we incubated with the primary antibodies overnight at 4 °C. The primary antibodies used were: rabbit-anti NAD-dependent deacetylase sirtuin-1 (SIRT1; 1:100, Millipore), rabbit anti-acetyl-Histone H3 (Lys9) (Acetyl H3-K9; 1:50, Millipore), rabbit anti-acetyl-p53 (Lys373), anti-growth associated protein-43 (GAP43; 1:50, Millipore), mouse anti-hypoxia inducible factor 1α (Hif-1 α; 1:200 amplified with streptavidin-biotin, Novus Biologicals), mouse anti-Atg5 (1:1000, Nanotools), and rabbit anti-phospho ribosomal protein S6 kinase (Thr 389) (p-P70S6K; 1:100, Antibodies Online). We washed with TBS-0.1% Tween-20 to remove the primary antibody excess and added specific donkey-Cy3 or Alexa 488 secondary antibodies (1:200; Jackson Immunoresearch) for 1 h and 30 min at RT. We then washed the slices with TBS-0.3% Triton-X-100 and added the fluorescent green NeuroTracer Nissl Stain (Molecular Probes, Leiden, Netherlands) and DAPI (Sigma, St Louis, MO, USA) to counterstain. After several washes with TBS and tris buffer, slices were mounted with Fluoromount-G mounting medium (SouthernBiotech). 

We examined with a Confocal Laser Scanning Microscope (Zeiss LSM 700; Zeiss, Jena) the immunolabeled spinal cords from different animals and experimental groups. Confocal images were systematically acquired using three separate photomultiplier channels with a 20x objective of 1.4 numerical aperture under the same conditions of exposure time, resolution, and sensibility for each analyzed marker. Images were separately projected and the signal intensity was analyzed with the aid of the ImageJ software (National Institutes of Health; available at http://rsb.info.nih.gov/ij/). The Nissl labeling was used as ROI (Region Of Interest) to enclose the MN cytosolic area, and the integrated density within the ROI was obtained for at least 15 MNs extracted from three different sections (separated 100 μm between each other) per animal for each marker [7].

### 2.9. Western Blotting

For immunoblotting studies, we collected the L4–L5 segment of the spinal cord from each animal (n = 3/4 per experimental condition) at end-stage and added lysis buffer (50 mM Tris, 2 mM EDTA, 0.5% Triton-X-100, 10 mM Nicotinamide, and a cocktail of protease [Sigma] and phosphatase [Roche] inhibitors; pH = 6.8). We homogenized lysates on ice with the aid of a pellet pestle (Sigma-Aldrich, sonicated it with an ultrasonic homogenizer (Model 3000, Biologics Inc) and we centrifuged at 13,000 g during 10 min at 4 °C. The supernatant was harvested and quantified with the BCA assay (Pierce Chemical Co.) according to the manufacturer’s instructions. Equal amounts of proteins from each animal (10 µg/well) were resolved on SDS-PAGE gels and transferred to a nitrocellulose membrane in a BioRad cuvette system in standard buffer composition (25 mM Tris, 192 mM glycine, 20% (v/v) methanol, pH 8.4). We blocked the proteins for 1 h at RT with 5% low fat milk in 0.1% Tween-TBS for 1 h at RT and incubated them overnight with different primary antibodies: rabbit anti LC3B (1:1000, Abcam), mouse anti-p62 (1:500, Novus Biologicals) mouse anti-p-ULK1 (1:500, Santa Cruz Biotechnology), mouse anti-Atg5 (1:1000, Nanotools), rabbit anti-Beclin1 (1:2000, Abcam), and anti-β-actin (Actin; 1:3000; Sigma Aldrich). After several washes, we incubated the membrane for 1 h with an appropriate secondary antibody conjugated with horseradish peroxidase (1:5000, Vector). The proteins were visualized using a chemiluminescent method (ECL Clarity kit, Bio-Rad Laboratories, Berkeley, CA, USA) and the images were captured and quantified with Image Lab Software (Bio-Rad Laboratories). 

### 2.10. Statistical Analysis

Data are presented as the mean ± standard error of the mean (SEM). Results were statistically analyzed using the GraphPad Prism 5 software (San Diego, CA, USA) by unpaired *t*-test to compare two groups, or one or two-way analysis of variance (ANOVA) followed by Bonferroni’s post hoc test between groups. Statistical significances were taken with a *p*-value of <0.05.

## 3. Results

### 3.1. ATG5 Overexpression Increases Functional Reinnervation and Recovery 

**Figure 1:**
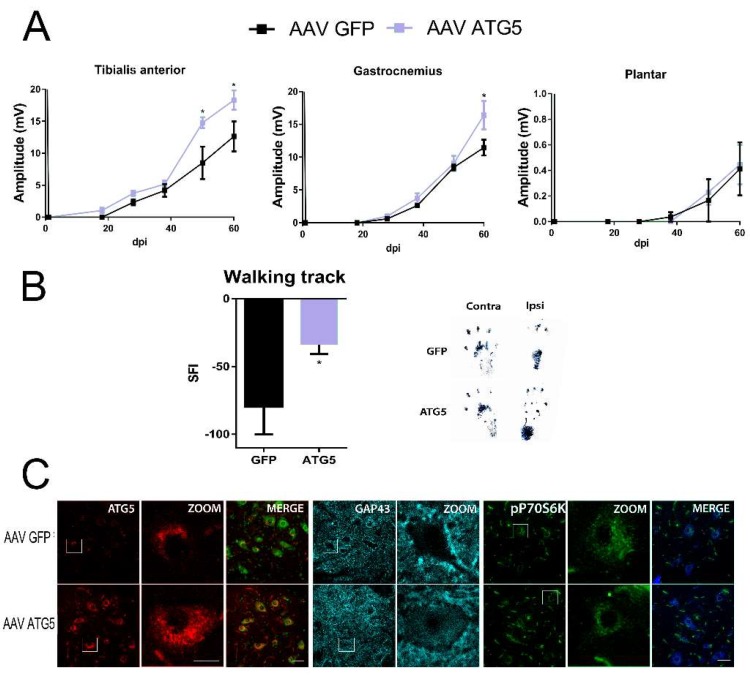
ATG5 overexpression increases motor axon regeneration. (**A**) Mean amplitudes (±SEM) values of compound muscle action potential (CMAP) recordings obtained during follow-up post-axotomy from tibialis anterior, gastrocnemius, and plantar muscles in animals overexpressing GFP or ATG5 (n = 4–5, ANOVA, post hoc Bonferroni, * *p* < 0.05 vs. AAV-GFP). (**B**) ***Left***, Graph of the sciatic functional index (SFI) obtained with the walking track analysis of the sciatic nerve in injured animals overexpressing ATG5 or GFP (*t*-test, * *p* <0.05 vs. AAV-GFP). ***Right***, representative footprints from ipsi- and contra-lateral paws at 60 days post-injury (dpi). (**C**) Representative confocal images of infected motoneurons (MNs) with either AAVrh10-GFP or AAVrh10-ATG5, immunolabeled for ATG5, GAP-43, and phospho-p70S6K at T-389, counterstained with FluoroNissl Green (left panels) or FluoroNissl Blue (right panels) and merged images at 60 dpi after nerve axotomy. Scale bar = 50 µm and 25 µm for ZOOM.

In order to ascertain whether autophagy induction was important in motor nerve regeneration, we aimed to investigate the potential to shift MNs to a growth-competent state by ATG5 overexpression, previously shown to induce autophagy [25]. For this purpose, a nerve conduction test was performed to rats injured with sciatic nerve transection and suture that received previously intrathecal injections of AAV vectors (AAVrh10) to overexpress either ATG5 (AAVrh10-ATG5) or the non-related protein GFP (green fluorescence protein) (AAVrh10-GFP), specifically within the spinal MNs [26]. The compound muscle action potential (CMAP) response of the gastrocnemius (GA), tibialis anterior (TA), and plantar interosseous muscles recorded at one week after nerve injury demonstrated complete denervation of the hindlimb muscles. Initial evidence of functional reinnervation of TA, GA, and plantar was found at around 18, 28, and 38 days post-injury, respectively, in some animals from all groups with CMAPs of small amplitudes. The amplitudes progressively increased with a similar pattern and magnitude in both group of animals. However, we detected higher TA and GA recorded amplitudes in the AAVrh10-ATG5 than in AAVrh10-GFP groups at 48 days (TA) and 60 days post injury (dpi) (TA and GA) (*p* < 0.05) (Figure 1A). No differences in functional reinnervation were observed more distally with still very small amplitudes (<0.05 mV) by 60 dpi for the plantar muscles. Importantly, we found a significant reduction of the sciatic functional index (SFI) analyzed by walking and tracking of the animals. This result suggested that the motor performance of hindlimb movements was better in the AAVrh10-ATG5 than in the AAVrh10-GFP group (Figure 1B).

We confirmed that by 60 dpi, ATG5 was still overexpressed within MNs of the AAVrh10-ATG5 group compared to the AAVrh10-GFP group of injured animals (Figure 1C). Furthermore, this was accompanied by the presence of abundant GAP43-positive staining within MNs suggesting a persistent growth-competent state in the former group (Figure 1C). Since we previously had demonstrated increased autophagy induced by ATG5 overexpression in MNs using this viral vector, we wondered whether this was dependent on mTOR kinase. To test this, we analyzed the levels of phosphorylated p70S6K (T-389), a widely mTOR kinase substrate used as a readout of mTOR activity [27,28], and found no differences between the two groups (Figure 1C). This observation pointed to the induction of mTOR-independent autophagy to improve functional reinnervation by ATG5 overexpression within MNs. 

Altogether, these results indicate that mTOR-independent autophagy activation is important for better motor regeneration and functional recovery. 

### 3.2. NeuroHeal Improves Axon Regeneration by SIRT1 Activation 

**Figure 2:**
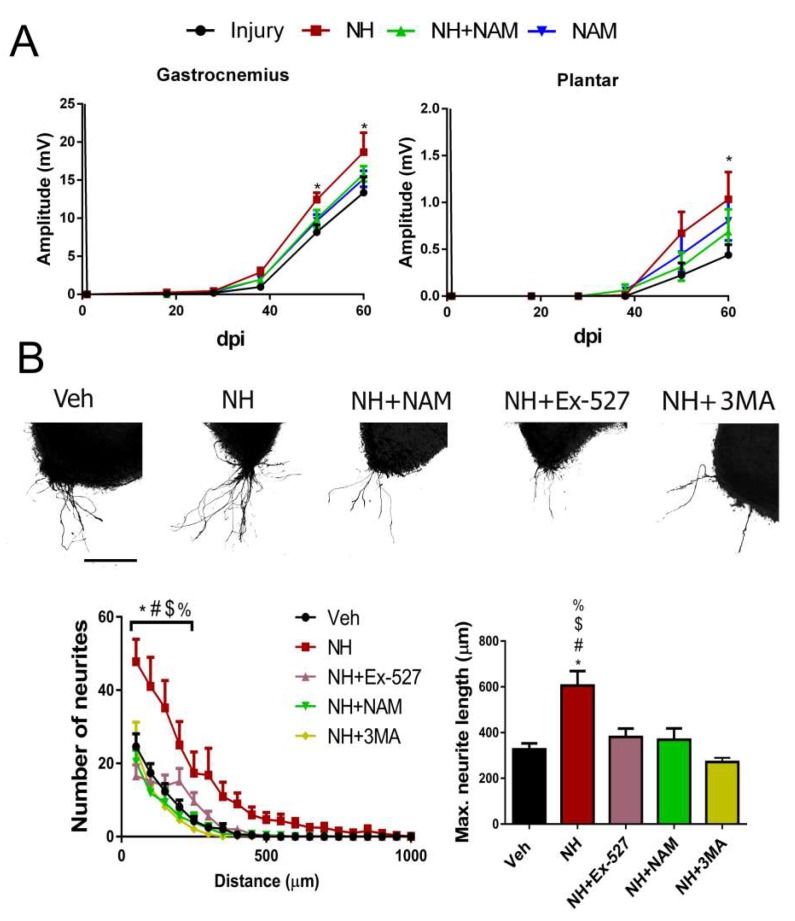
Pro-regenerative effect of NeuroHeal requires SIRT1 activity and autophagy. (**A**) Mean amplitudes (±SEM) values of CMAP recordings obtained during follow-up post-axotomy from gastrocnemius (GA) and plantar muscles in animals treated with NH, NH+nicotinamide (NAM), or NAM (n = 5–6, ANOVA, post hoc Bonferroni, * *p* < 0.05 vs. Injury). (**B**) Representative microphotographs of Veh-, NH-, NH+Ex-527-, NH+NAM-, and NH+3MA-treated spinal cord organotypic cultures (SOCs) embedded in collagen. Graphs show the number of neurites per intersection and the maximum neurite length in the SOC (n = 8–10, ANOVA, post hoc Bonferroni, * *p* < 0.05 vs. Veh, ^#^ *p* < 0.05 vs. NH+Ex-527, ^$^ *p* < 0.05 vs. NH+NAM, ^%^ *p* < 0.05 vs. NH+3MA). Scale bar = 250 µm.

Considering that autophagy can be triggered by AMPK as an mTOR independent mechanism [29], we investigated whether downstream SIRT1 activation that regulates the formation of autophagic vacuole may also improve regeneration [30]. We first performed a pharmacologic approach using the previously described NeuroHeal, as a SIRT1 activator [7,18]. The NeuroHeal-treated group of rats with cut and suture of the sciatic nerve presented significantly higher CMAP amplitudes compared to the vehicle-treated group of the GA muscle at 48, and 60 dpi, and of the plantar muscle at end-point (*p* < 0.05, Figure 2A). Interestingly, these differences were lost in injured animals treated with NeuroHeal plus nicotinamide (NAM), which may act as SIRT1 inhibitor (Figure 2A). Using a different model, 3D collagen matrix embedded spinal cord organotypic cultures (SOCs) [23], we assessed facilitation of neurite outgrowth after NeuroHeal treatment alone or in combination with two well-known inhibitors of SIRT1 activity, Ex-527, and NAM. NeuroHeal treatment significantly increased the number and maximum length of neurites propelled out from SOCs within the permissive substrate (Figure 2B). We verified that the effect stimulated by NeuroHeal could not be attributed to any of its single components, acamprosate or ribavirin (Figure S1). The concomitant treatment of NeuroHeal with the SIRT1 inhibitors completely blocked its pro-neuritogenic effect and the maximum length of the extended neurites. In agreement with this, the level of GAP43, a hallmark of regeneration, was only increased by NeuroHeal treatment but was downregulated when adding SIRT1 inhibitors (Figure S2). These results suggest that SIRT1 activation by NeuroHeal facilitates its pro-regenerative effect. 

To investigate whether autophagy induction had a role in neurite outgrowth mediated by SIRT1 activation, we treated SOCs with the PI3K inhibitor 3-Methyladenine (3MA) that inhibits autophagy by blocking autophagosome formation via the inhibition of class III phosphoinositide 3-kinase (PI3K/hVps34) when used in short periods to inhibit autophagy [31]. PI3K mediates autophagy at both the initiation and maturation stages of autophagosomes [31]. We observed that the addition of 3MA on NeuroHeal-treated SOCs blocked its beneficial effects on both neurite outgrowth and elongation (Figure 2B). 

### 3.3. Autophagy Promoted by SIRT1 Overexpression Is Necessary for Nerve Regeneration 

**Figure 3:**
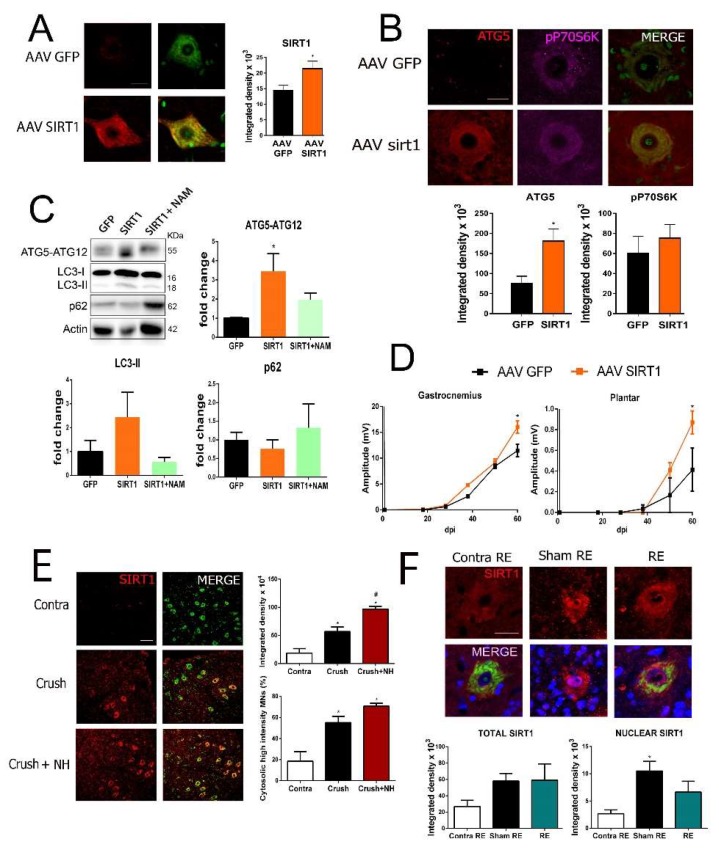
NAD^+^-dependent deacetylase sirtuin-1 (SIRT1) promotes autophagy in MNs and is cytosolic during motor axon regeneration. (**A**) Representative confocal images of SIRT1 (red) counterstained with FluoroNissl (green) in MNs from the AAVrh10-GFP or AAVrh10-SIRT1 after axotomy at 60 dpi Scale bar = 20 µm. (**B**) Representative confocal images of MNs stained with ATG5 and p-p70S6K at T-389 with FluoroNissl green from the different groups and associated bar graphs of the mean (±SEM) intensity for each marker inside the cytoplasm of injured MNs at 7 dpi (n = 4 animals per group, *t*-test, * *p* < 0.05 vs. AAV-GFP) Scale bar = 20 µm. (**C**) Western blot and corresponding bar graphs of the quantification of different proteins related to autophagy (ATG5, LC3-II, and p62) in the spinal cord from axotomy-injured animals injected with AAVrh10-GFP or AAVrh10-SIRT1 with or without oral nicotinamide (NAM). (n = 4, ANOVA, post hoc Bonferroni, * *p* < 0.05 vs. AAV-GFP). (**D**) Mean amplitudes (±SEM) values of CMAP recordings obtained during follow-up post-axotomy from the gastrocnemius (GA) and plantar muscles in animals overexpressing GFP or SIRT1 (n = 4–6, ANOVA, post hoc Bonferroni, *p < 0.05 vs. AAV-GFP (**E**) ***Up***, Confocal images of MNs immunolabeled for SIRT1 in red and counterstained with FluoroNissl (green) and DAPI (blue) from MNs of Contra, crush, and NH-treated animals at 35 dpi. Scale bar = 50 µm. ***Down***, Histograms of the mean of the SIRT1 immunofluorescence intensity inside the cytoplasm of injured MNs (n = 3 animals per group, ANOVA, post hoc Bonferroni, * *p* < 0.05 vs. contra and ^#^ *p* < 0.05 vs. Crush). (**F**) ***Top***, Confocal microphotographs and graphs showing levels of SIRT1 (red) co-labeled with FluoroNissl (green) from contralateral site (Contra RE), sham (Sham RE), and after peripheral nerve root avulsion with reimplant (RE). Scale bar = 25 µm. ***Bottom***, Graph of the means of the immunofluorescence intensity inside MNs or inside the nuclei of MNs (n = 3–4 per group, ANOVA, post hoc Bonferroni, * *p* < 0.05 vs. Contra RE, ^#^ *p* < 0.05 vs. Sham RE).

We further characterize whether specific SIRT1 overexpression may induce autophagy and increase functional nerve reinnervation. We generated AAVrh10-SIRT1 particles to drive its expression into spinal MNs in animals posteriorly subjected to microsurgery for cut and suture of the sciatic nerve. As expected, SIRT1 expression increased in the cytosol of spinal MNs in animals injected with AAVrh10-SIRT1 compared to those with AAVrh10-GFP at 7 dpi (Figure 3A). One key molecule in autophagy is ATG5, which, when conjugated to ATG12, forms part of the complex that mediates the lipidation of Microtubule-associated protein 1 light chain 3 (MAP-LC3/Atg8/LC3 II) at the autophagosome [32,33]. We observed that ATG5 was accumulated in damaged MNs when SIRT1 was overexpressed compared to the AAVrh10-GFP group (Figure 3B). In contrast, the level of the phosphorylated form of p70S6K (T-389) that depends on mTOR-activity did not differ between the two groups (Figure 3B). We also analyzed the level of some autophagy markers by immunoblotting. At 7 dpi, we observed a significant increase in the level of ATG5-ATG12-conjugate and a trend of an increase for the LC3II isoform, promoted by SIRT1 overexpression compared to the AAV10-GFP group (Figure 3C). No differences were found in p62 levels, whose accumulation usually indicates autophagy flux blockade. These effects were reversed by treatment with NAM in animals from the AAVrh10-SIRT1 group where only p62 levels had a tendency to increase (Figure 3C).

Extended follow up of the animals allowed for CMAP amplitudes recording. We found that animals from the AAVrh10-SIRT1 group presented significant higher amplitudes than those in the AAVrh10-GFP group, in both GA and plantar muscles by 60 dpi (Figure 3D). Altogether, these results suggest that SIRT1 overexpression and activation have a relevant role in promoting motor nerve regeneration.

### 3.4. SIRT1 Is in the Cytosol of Spinal MNs in Growth-Competent State

To investigate further the underlying mechanisms downstream SIRT1-mediated autophagy important for nerve growth, we considered the observation that SIRT1 was mainly located at the cytosol within MNs in the AAVrh10-SIRT1 group, while its normally reported location is in the nucleus of neurons. This was not a single event since we also found SIRT1 within the cytosol in other models. For instance, after sciatic nerve crush, MNs switch from a transmitting mode to a growth-competent state as a pro-regenerative mode [34]. We observed an increase in cytosolic SIRT1 within MNs, already after nerve crush and when the injured animals were treated with NeuroHeal, which also improved regeneration in this model, as previously reported [18] (Figure 3E). In another model of ventral root avulsion and delayed reconnection [6], we had also observed that SIRT1 was translocated from the nucleus to the cytosol only when reconnection was allowed (Figure 3F). These observations suggested that SIRT1 activity should be enhanced in the cytosol to allow nerve regeneration.

### 3.5. SIRT1/HIF1α-Autophagy Axis Activation Enhances Neurite Outgrowth

**Figure 4:**
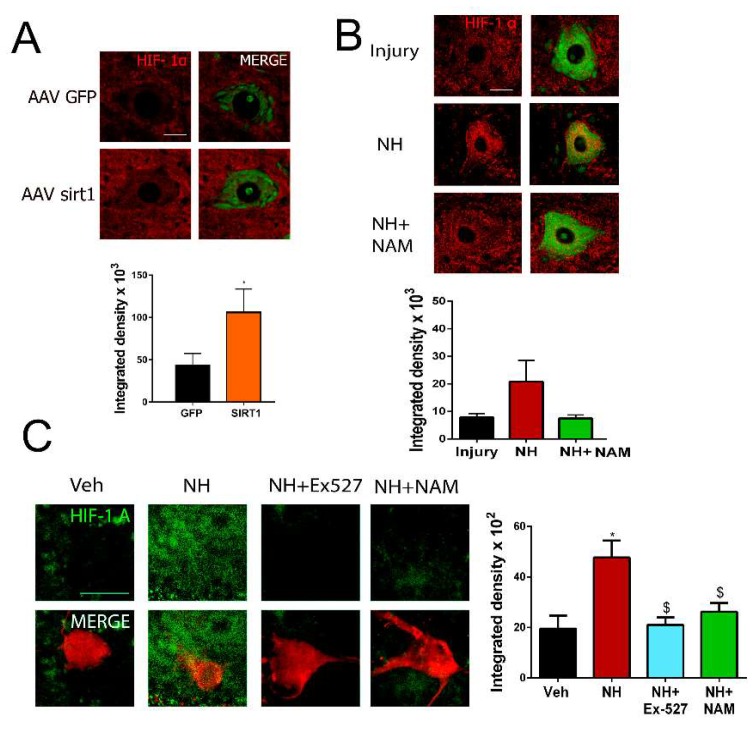
SIRT1 activity stabilizes HIF1α in motoneurons (**A**) Representative confocal images of MNs stained with HIF1-α FluoroNissl green from the different groups and associated bar graphs of the mean (±SEM) intensity for each marker inside the cytoplasm of injured MNs from AAVrh10-GFP or AAVrh10-SIRT1 animals. (n = 4 animals per group, *t*-test, * *p* < 0.05 vs. AAV-GFP) Scale bar = 20 µm. (**B**) Representative confocal images of Hif-1α (red) counterstained with FluoroNissl (green) in MNs from the different groups at 60 dpi. Scale bar = 20 µm ***Bottom***, a bar graph of the mean (±SEM) intensity for Hif1-1α inside the cytoplasm of injured MNs at 60 dpi (n = 3–4, ANOVA, post hoc Bonferroni). Scale bar = 20 µm (**C**) ***Left***, Representative confocal images of HIF-1α (green) counterstained with RT-97 (red) of MNs from SOCs of different conditions. Scale bar = 20 µm. ***Right***, Bar graphs of the mean (±SEM) intensity for each marker inside the cytoplasm of MNs (n = 10–18, ANOVA, post hoc Bonferroni, * *p* < 0.05 vs. Veh, ^$^ *p* < 0.05 vs. NH).

Regarding the presence of SIRT1 in the cytosol, we explored changes in one of its cytosolic substrates, the hypoxia-inducible factor 1α (HIF-1 α), because of its reported relationship with regeneration in *Caenorhabditis elegans* and sensory neurons [35,36,37]. We observed that Hif1α was accumulated in AAVrh10-SIRT1 compared to AAVrh10-GFP group of injured animals by nerve cut and suture (Figure 4A). Similarly, injured animals treated with NeuroHeal presented higher levels of Hif1α than vehicle-treated animals in both the cut/suture model (Figure 4B) and the crush model (Figure S3). Likewise, in SOC explants, the addition of NeuroHeal increased the levels of Hif1α, which was blocked by the concomitant addition of SIRT1 inhibitors EX527 or NAM (Figure 4C).

**Figure 5:**
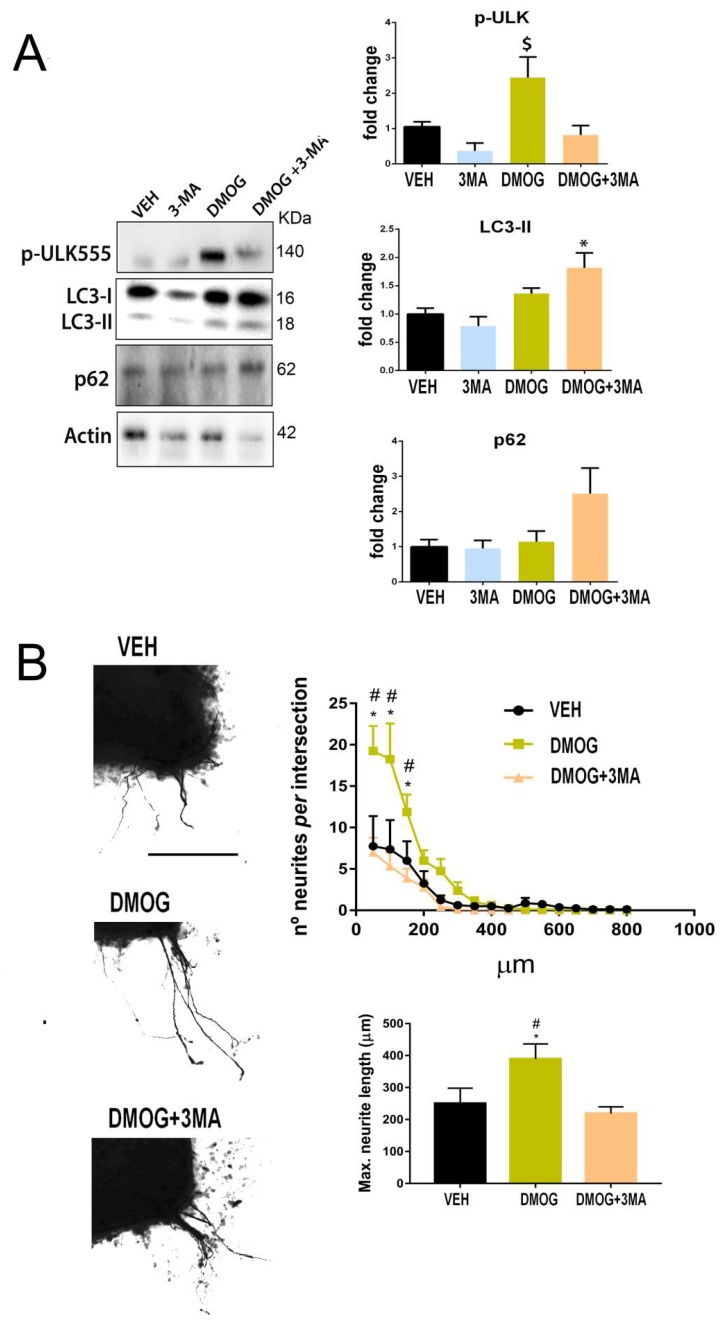
Hif-1α-dependent autophagy increases motor axon growth. (**A**) Western blots and histogram showing the analysis of pUlk1 (Ser555), ATG5-ATG12 LC3II, and p62 protein level from SOCs after 2 days of treatment with vehicle (Veh), DMOG, 3-MA, or DMOG+3-MA (n = 3–4, ANOVA, post hoc Bonferroni * *p* < 0.05 vs. DMOG, ^#^ *p* < 0.05 vs. Veh). (**B**) Representative microphotographs of Veh-, DMOG-, and DMOG+3MA-treated SOCs embedded in collagen. Graphs show the number of neurites per intersection and the maximum neurite length in the SOCs (n = 8–9, ANOVA, post hoc Bonferroni, * *p* < 0.05 vs. Veh, ^#^ *p* < 0.05 vs. DMOG+3MA). Scale bar = 250 µm.

Considering the studies available in the literature that correlate either the cytosolic activity of SIRT1 or HIF-1 α with autophagy [38,39,40,41], we aimed to further explore this pathway in the regenerative context. To investigate whether Hif1α-induced autophagy has pro-regenerative effects, we treated SOCs with DMOG, an inhibitor of the prolyl hydroxylases, enzymes that drive Hif1α degradation by hydroxylation [42]. We had previous evidence that HIF1α was stabilized after DMOG treatment in a model of hypoglossal nerve injury (Figure S4). We observed that DMOG treatment of SOCs augmented pUlk1 (Ser555) levels, a marker of autophagy induction, although no differences were observed in LC3II or p62 levels (Figure 5A). The addition of 3MA impeded pUlk1 increase by DMOG as expected, but increased LC3II and p62 levels suggesting autophagy flux blockage (Figure 5A). We also analyzed neurite outgrowth and observed that SOCs treated with DMOG significantly boosted neurite outgrowth and length, which was abolished by the addition of 3MA (Figure 5B). These results suggest that Hif1α accumulation has a neuritogenic effect that depends on autophagy.

**Figure 6:**
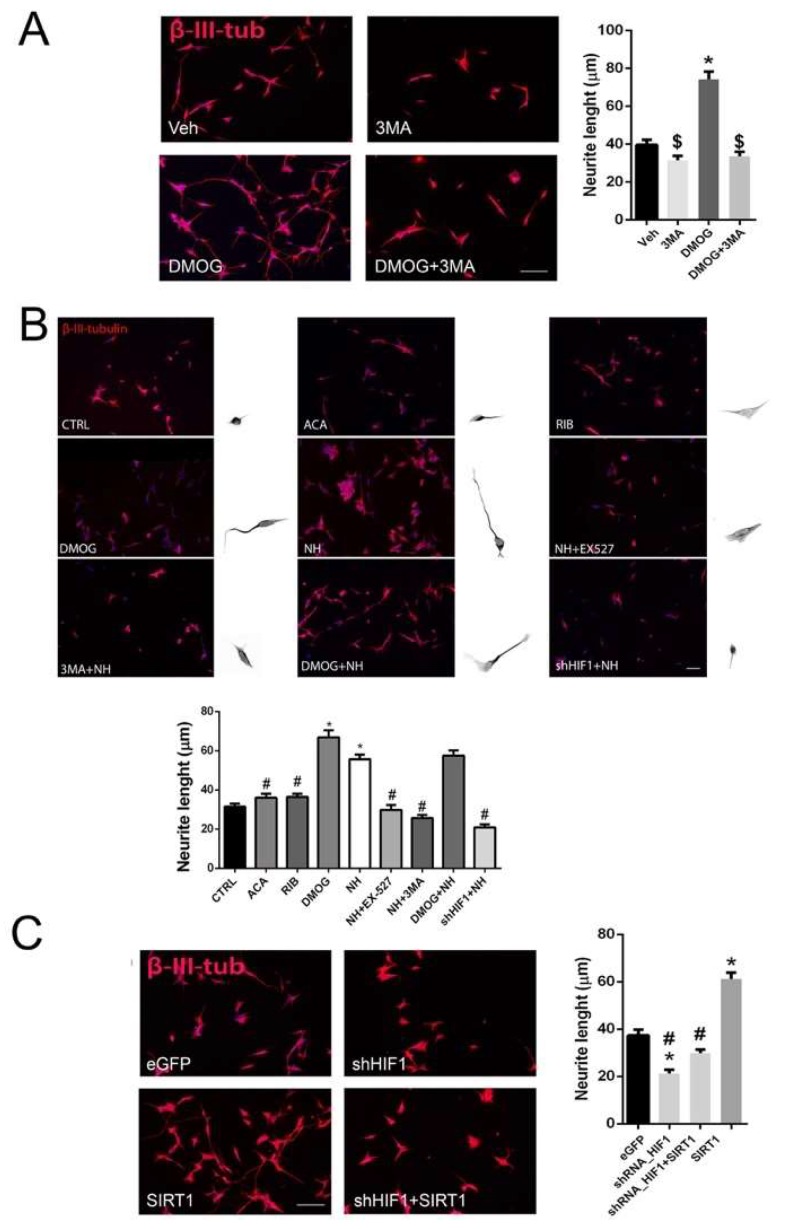
SIRT/HIF1α-autophagy axis activation enhances neurite outgrowth. (**A**) Representative microphotographs and bar graphs showing the mean (±SEM) values of neurite length from SH-SY5Y cells after 24 h of treatment with Vehicle, DMOG, 3-MA, or DMOG+3-MA on transfected cells. (n = 3–4, ANOVA, post hoc Bonferroni * *p* < 0.05 vs. Veh, ^$^ *p* < 0.05 vs. DMOG). Scale bar = 100 µm. (**B**) ***Top***, Microphotographs and representative neurons outset 300X magnification. ***Bottom***, bar graphs showing the mean (±SEM) values of neurite length from SH-SY5Y in different experimental groups. (n = 3–4, ANOVA, post hoc Bonferroni * *p* < 0.05 vs. Veh, ^#^ *p* < 0.05 vs. NH). Scale bar = 100 µm. (**C**) Representative microphotographs and bar graphs showing the mean (±SEM) values of neurite length from SH-SY5Y cells in transfected cells with eGFP, shRNA/HIF1, SIRT-1 or the combination of shRNA/HIF+ SIRT-1. (n = 3–4, ANOVA, post hoc Bonferroni * *p* < 0.05 vs. eGFP, ^#^ *p* < 0.05 vs. SIRT1). Scale bar = 100 µm.

To confirm whether the SIRT1 pro-regenerative effect was mediated by Hif1α stabilization, we used another in vitro model based on the neurite extension observed in the human cell line SH-SY5Y. We observed that DMOG treatment significantly increased the average neurite length extended out from these cells as observed in SOCs and analyzed with β-III-tubulin immunostaining (Figure 6A). Similarly to what observed in SOCs, the addition of 3MA to the DMOG-treated cells prevented these effects (Figure 6A). NeuroHeal treatment also increased neurite length, an effect that was abolished by SIRT1 inhibition with EX527 or autophagy inhibition with 3MA (Figure 6B). Furthermore, we nucleofected the cells to overexpress SIRT1 and/or silence Hif1α using shRNA (Figure S5). We observed that SIRT1 overexpression significantly increased average neurite length, which was abolished by Hif1α shRNA, indicating that Hif1α was crucial in promoting the SIRT1-dependent axonal regeneration. (Figure 6C).

## 4. Discussion

In the present work, we aimed to explore whether autophagy induction was an important issue in favor of motor nerve regeneration after PNI. We found that specific induction of autophagy by ATG5 or SIRT1 overexpression improved motor functional reinnervation. Interestingly, most of the SIRT1 overexpression was located in the cytosol of growth-competent state MNs narrowing the possible downstream substrates. Among these, HIF1α stabilization and downstream activation of autophagy was found necessary to promote neurite outgrowth in organotypic spinal cord cultures and human cells.

We have found that activation of autophagy, by either ATG5 or SIRT1 overexpression, improves motor functional reinnervation in a rat model of nerve transection and repair. We observed an increase in the ATG5-ATG12 complex in the spinal cord from SIRT1-overexpressing animals suggesting that the origin of autophagy was in the MN soma as a driving force of motor nerve regeneration. A lesion triggers a drastic response in soma and axon, including chromatolysis, axonal membrane sealing, growth cone Rohon–Beard (RB) formation, and Wallerian degeneration. The growth cone initiation and axon regeneration require extensive remodeling of the cytoplasmic compartment and axon structures, which involve the synthesis and degradation of local proteins. Autophagy is one of the major pathways for bulk cytosolic degradation and efficient turnover under stress. Our study is in agreement with He and collaborators (2016), who reported induced autophagy promotes central nervous system axonal regeneration after injury by increasing microtubule (MT) stability [16]. In addition, we previously observed that ATG5 overexpression in MNs induced autophagy but also corrected cytoskeletal alterations [25]. The fact that plantar CMAP amplitudes are significantly greater within 60 days when overexpressing SIRT1 than when overexpressing ATG5, suggests that other pathways triggered by SIRT1 might be synergistically contributing to a better recovery.

SIRT1 has been reported to play a central role in axonogenesis and optic nerve regeneration [43,44,45,46,47]. In sensory axonal regeneration, SIRT1 activation slows its degeneration from dorsal root ganglia [48]. Herein, we show that SIRT1 activation or overexpression improves motor nerve regeneration after damage. Particular interesting is the fact that SIRT1’s main cytosolic location correlates with a growth-competent state of the MN in several animal models of PNI. Although SIRT1 is predominantly a nuclear protein, a similar cytosolic location was found in neonatal and adult MNs after injury and after NeuroHeal treatment [7,18]. Other authors have reported SIRT1 in the cytosol related to the process of neuronal differentiation [49,50,51]. We argued that this might be related to the availability of nicotinamide adenine dinucleotide (NAD^+^), necessary to sustain SIRT1 deacetylase enzymatic activity in specific organelles [52]. Local NAD^+^ production may be strictly modulated by recruitment of NAD^+^-biosynthetic enzymes to sites of NAD^+^-consuming reactions [53]. NAD^+^ synthesis is independently regulated in the nucleus and the cytosol, and the cytoplasmic NAD^+^ pool is maintained primarily by the NAD^+^-synthesizing enzyme nicotinamide mononucleotide adenylyltransferase (NMNAT). Synthesis and maintenance of high NAD^+^ concentrations have been considered crucial to axon integrity [54] and SIRT1 is an effector of the axonal protection mediated by increased NMNAT1 activity, whose location might be nuclear or cytosolic. However, it was recently shown that the activity of NMNAT1 was both necessary and sufficient to prevent axonal degeneration only when translocated to cytoplasmic compartment [55,56]. The treatment with NeuroHeal might produce abundance of cytosolic NAD^+^ since it inhibits IMPDH, a protein largely cytosolic that catalyzes the NAD^+^-dependent oxidation of IMP to xanthosine monophosphate [18,57]. Hence, particular cytosolic overexpression of SIRT1 might be a key factor in promoting nerve regeneration and deserves further studies. From the therapeutic point and a view towards precision medicine, these observations highlight that it is not only important to activate or inhibit a specific target, like SIRT1, but also its activity within a particular subcellular compartment.

In this study, we attempted to unravel SIRT1 downstream signaling involved in nerve regeneration by analyzing Hif1α, one of its cytosolic substrates, since SIRT1 deacetylates and stabilizes it in this compartment [58]. Previous studies demonstrated Hif1α was implicated in neuritogenesis in *C. elegans* and in mammalian sensory nerve regeneration [36,37,59]. Herein, we confirm Hif1α’s important role in neuritogenesis within the SIRT1/Hif1α axis. The question that remained is how? HIF-1 is a transcriptional mediator of the cellular response to hypoxia, a form of cellular stress. HIF-1 is a heterodimeric transcription factor consisting of two subunits, HIF-1α and HIF-1β [60,61]. One possibility examined in the present work considered that HIF1α induced downstream mTOR-independent autophagy as it has been previously reported [62]. This was interesting since previous work pointed to local autophagy having an important role in preventing axonal degeneration or promoting axonal growth. For instance, a recent study showed that Ulk induces axonal guidance [63] or, after spinal cord injury, the autophagy induction avoids the retraction of central nervous system axons and enhances their regrowth leading to an increased recovery of motor function [16]. Although it is known that SIRT1 may drive directly autophagy by inhibiting mTORC1 and FOXO, and activating key regulatory proteins, such as ATG5, ATG7, and LC3 [52], we have demonstrated herein that HIF1a is necessary for SIRT1-mediated neuritogenesis. Interestingly, there are other non-genetic activities for HIF1a in the cytosol that might be relevant to autophagy and axon regeneration. Cytosolic HIF1a might bind the chaperone Hsp90 [64] and Dicer [65]. It is known that HIF1a prevents Hsp90 translocation to the nucleus although it is unknown the repercussion on its necessity for proper neuronal polarization and axon elongation [66]. Another relevant partner is Dicer, which mediates ubiquitination and autophagic proteolysis and might bridge HIF1α to p62 [65]. In conclusion, several routes are now being explored to provide further details about the components involved in autophagy-pro-regenerative capability.

### Study Limitations and Future Research

The first limitation of this study was that autophagy was analyzed in a particular fixed time-window. Autophagy flux is a dynamic process and, thus, it would be interesting to unravel the implication of our pharmacological and genetic modifications in the flow and the molecules and processes involved. However, due to the largely different experimental groups, we had to focus on a specific time-point post-injury. Another limitation was that we only analyzed motor axon regeneration, not considering the effects of NeuroHeal in sensory neurons. We are now initiating these studies in our lab. Indeed, the work presented here has opened new research lines. We are currently determining the analgesic effects of NeuroHeal after PNI to reduce neuropathic pain, based on recent articles suggesting that autophagy is involved in nociception [67,68]. Moreover, a recent article demonstrates that autophagy induction promotes recovery after spinal cord injury by clearance of an axonal-growth inhibitory protein [16]. Therefore, SIRT1/Hif1α-mediated autophagy might enhance functional recovery after spinal cord injury. All these novel research topics will be explored in the near future.

## 5. Conclusions

In summary, we demonstrate that SIRT1 activation is important for axonal regeneration and that Hif1a and downstream autophagy mediates this action; thus, our work has identified a new endogenous mediator of axon regeneration in MNs. Altogether, the knowledge advanced from the present study opens avenues for therapeutic precise options for nerve repair after PNI.

## 6. Patents

NeuroHeal is currently under patent review.

## Supplementary Materials

The following Figures (S1–S5) are available online at https://www.mdpi.com/2073-4409/8/11/1354/s1.
