# Supplementary material for: Improved Motor Nerve Regeneration by SIRT1/Hif1a-Mediated Autophagy
Source: Cells. 2019 Oct 30;8(11):1354. doi: 10.3390/cells8111354 (PMC6912449; doi:10.3390/cells8111354)
Supplement: Supplementary file 1 [file cells-08-01354-s001.pdf]

Fig. S1.

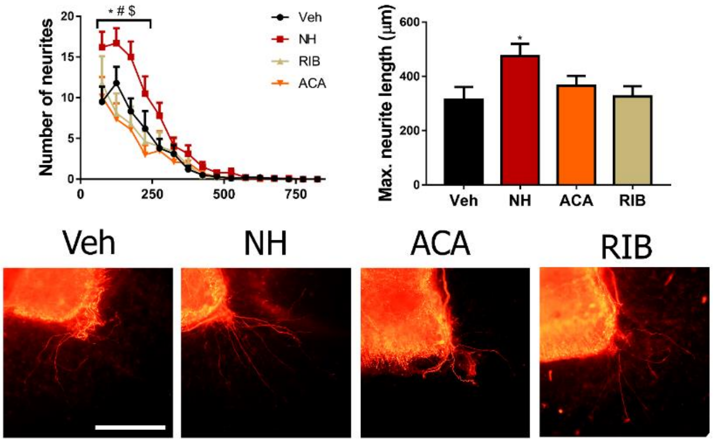

NeuroHeal presents a synergistic effect promoting motor axon growth. Representative microphotographs of Veh-, NH-, ACA-, and RIB-treated SOCs embedded in collagen. Graphs show the number of neurites per intersection and the maximum neurite length in the SOC (n=8-9, ANOVA, post hoc Bonferroni, *p < 0.05 *vs.* Veh, # *p* < 0.05 *vs.* ACA and $ *p*<0.05 *vs.* RIB). Scale bar = 250 µm.

Fig. S2.

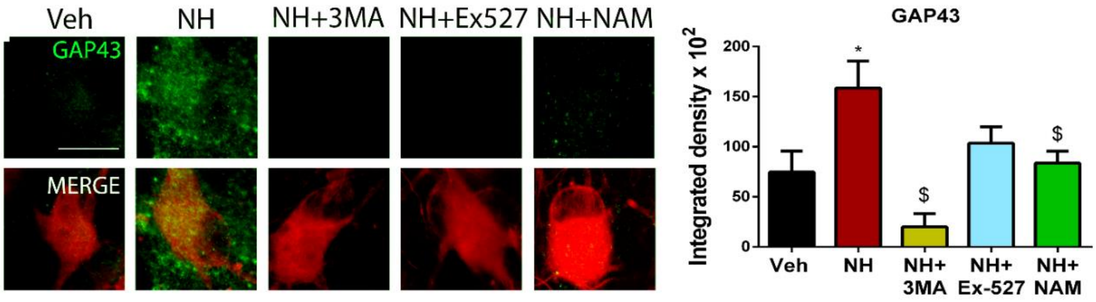

NeuroHeal increases GAP43 presence. ***Left***, Representative confocal images of immunostaining for GAP43 (green) and for neurofilament 200 kDa (NF-H) (red, RT-97 antibody) from MNs in different experimental groups. ***Right***, a bar graph of the mean (±SEM) intensity of GAP43 in MNs of Vehicle (Veh)-, NeuroHeal (NH)-, NH+EX-527-, NH+3MA- and NH+NAM- treated SOCs (n=14-29, ANOVA, post hoc Bonferroni, *p<0.05 *vs.* Veh, $ p <0.05 *vs.* NH). Scale bar = 20 µm.

Fig. S3.

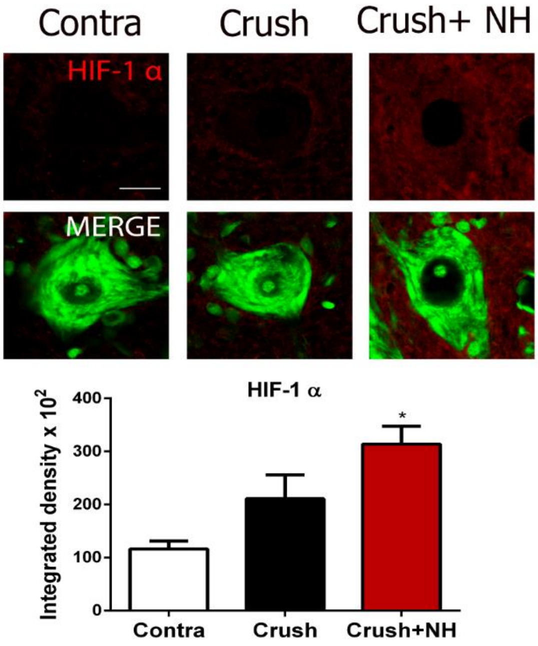

Hif-1α is increased after nerve injury in motoneurons. ***Up,*** Representative confocal images of Hif-1α (red) counterstained with FluoroNissl (green) in motoneurons (MNs) from the different groups at 60 dpi. Scale bar = 20 µm. ***Bottom,*** a bar graph of the mean (±SEM) intensity for Hif1-1α inside the cytoplasm of injured MNs at 60 dpi (n=3-4, ANOVA, post hoc Bonferroni, *p<0.05)**.**

Fig. S4.

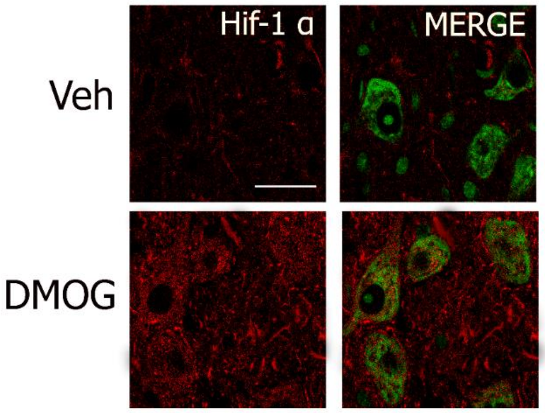

DMOG treatment stabilizes Hif-1α in motoneurons. Representative confocal images of injured motoneurons in a hypoglossal model immunolabeled for HIF1-α and counterstained with FluoroNissl Green at 21 dpi in injured animals and DMOG-treated animals. Scale bar =25 µm.

Fig. S5

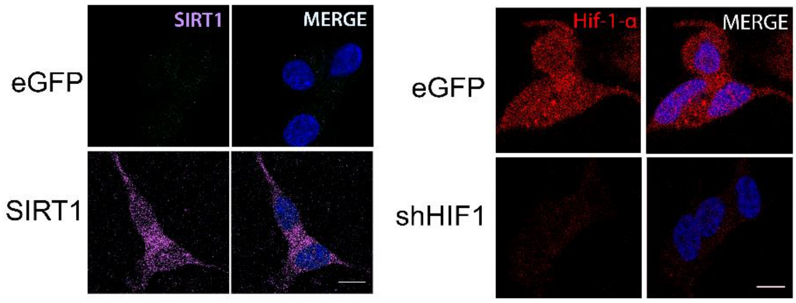

Effective SIRT1 overexpression or HIF1 silencing in SH-SY5Y. ***Left,*** Confocal microphotographs of SIRT1 in eGFP or SIRT1-transfected SH-SY5Y cells. ***Right,*** Immunocytochemistry against Hif-1α (red) in eGFP or shRNA/HIF1 transfected SH-SY5Y cells counterstained with DAPI. Scale bar = 25 μm.
